# **Triflumizole Is an Obesogen in Mice that Acts through Peroxisome Proliferator Activated Receptor Gamma (PPAR**γ**)**

**DOI:** 10.1289/ehp.1205383

**Published:** 2012-10-22

**Authors:** Xia Li, Hang T. Pham, Amanda S. Janesick, Bruce Blumberg

**Affiliations:** 1 Department of Developmental and Cell Biology, and; 2 Department of Pharmaceutical Sciences, University of California, Irvine, Irvine, California, USA

**Keywords:** 3T3-L1 cells, adipogenesis, endocrine disruption, MSCs, obesogen, PPARγ, triflumizole

## Abstract

Background: Triflumizole (TFZ) is an imidazole fungicide used on many food and ornamental crops. TFZ is not thought to be particularly toxic or carcinogenic, but little is known about its effect on development. TFZ is identified as a peroxisome proliferator activated receptor gamma (PPARγ) activator in ToxCast. Because PPARγ is a master regulator of adipogenesis, we hypothesized that TFZ would activate PPARγ, thereby inducing adipogenesis and weight gain *in vivo*.

Objectives: We sought to test the ability of TFZ to activate PPARγ and promote adipogenesis *in vitro* and *in vivo*.

Methods: We used transient transfection to test the ability of TFZ to activate PPARγ, and we used 3T3-L1 preadipocytes and human multipotent mesenchymal stromal stem cells (MSCs) to study the adipogenic capacity of TFZ in culture. We treated pregnant mice with three doses of TFZ and evaluated the effects on body weight, adipose depot weight, and MSC programming in the prenatally exposed offspring.

Discussion: TFZ induced adipogenesis in MSCs and in mouse 3T3-L1 preadipocytes. Prenatal exposure to levels of TFZ at approximately 400-fold below the reported no observed adverse effect level increased adipose depot weight. All doses of TFZ tested increased adipogenic gene expression in MSCs while inhibiting expression of osteogenic genes.

Conclusions: TFZ acts through a PPARγ-dependent mechanism to induce adipogenic differentiation in MSCs and preadipocytes at low nanomolar concentrations. Prenatal TFZ exposure increases adipose depot weight and diverts MSC fate toward the adipocyte lineage; therefore, we conclude that TFZ is an obesogen *in vivo*.

Obesity and related disorders are a public health epidemic, particularly in the United States. Currently > 34% of the U.S. population is clinically obese [body mass index (BMI) > 30] and 68% are overweight (BMI > 25), more than double the worldwide average and 10-fold higher than Japan and South Korea (Flegal et al. 2010). Genetics (Herbert 2008) and behavioral factors such as smoking (Power and Jefferis 2002), stress (Garruti et al. 2008), sedentary lifestyle (Rippe and Hess 1998), and excessive consumption of food (Hill and Peters 1998) are the typically cited causes of obesity. An alarming recent trend is the increasing rate of obesity in very young children, even infants (Koebnick et al. 2010; McCormick et al. 2010; Taveras et al. 2009). It is unlikely that infants are consuming more calories and exercising less than in the past, so it is reasonable to hypothesize that the prenatal and/or early postnatal environment has recently changed. Intriguingly, a recent study showed that animals [pets (cats, dogs), laboratory animals (rats, mice), four species of primates, and feral rats] living in proximity to humans in industrialized societies exhibited pronounced increases in obesity over the past several decades (Klimentidis et al. 2010). The likelihood of 24 animal populations from eight different species all showing a positive trend in weight over the past few decades by chance was estimated at 1 in 12 million (1.2 × 10^–7^) (Klimentidis et al. 2010). It is more probable that changes in one or more environmental components are making these animals obese in parallel with humans.

We proposed the existence of endocrine disrupting chemicals that could influence adipogenesis and obesity and be important, yet unsuspected, players in the obesity epidemic (Janesick and Blumberg 2011a). These “obesogens” are chemicals that promote obesity by increasing the number of fat cells or the storage of fat into existing cells. Obesogens can act indirectly by changing basal metabolic rate, by shifting the energy balance to favor calorie storage, and by altering hormonal control of appetite and satiety (Heindel 2011; Janesick and Blumberg 2011a; La Merrill and Birnbaum 2011; Newbold 2011). Several obesogenic chemicals have been identified in recent years, underscoring the relevance of this new model. Estrogens such as diethylstilbestrol (DES) (Newbold et al. 2009) and bisphenol A (BPA) (Rubin 2011; Rubin et al. 2001), organotins such as tributyltin (TBT) (Grun et al. 2006; Kirchner et al. 2010), and perfluorooctanoates (Hines et al. 2009) are obesogenic in animals. Phthalates were correlated with increased waist diameter (Hatch et al. 2008; Stahlhut et al. 2007), and high levels of several persistent organic pollutants (e.g., DDE, hexachlorobenzene, polybrominated diphenylethers) were linked with obesity in humans (Tang-Peronard et al. 2011). Relatively little is known about how many commonly used chemicals (e.g., industrial chemicals, pesticides) can act as obesogens *in vivo*.

TBT is a well-characterized obesogen that is a nanomolar affinity ligand for two nuclear receptors critical for adipocyte development: the 9-*cis*-retinoic acid X receptor (RXR) and peroxisome proliferator activated receptor gamma (PPARγ) (Grun et al. 2006; Kanayama et al. 2005). TBT promoted adipogenesis in murine 3T3-L1 preadipocytes (Grun et al. 2006; Kanayama et al. 2005) and in human and mouse multipotent mesenchymal stromal stem cells (MSCs, or mesenchymal stem cells) via a PPARγ-dependent pathway (Kirchner et al. 2010; Li et al. 2011). *In utero* TBT exposure led to strikingly elevated lipid accumulation in adipose depots, liver, and testis of neonate mice and increased adipose depot mass in adults (Grun et al. 2006). Because PPARγ is considered a master regulator of adipogenesis (Tontonoz and Spiegelman 2008), it is likely that other PPARγ activators will also prove to be obesogenic (Janesick and Blumberg 2011b).

The U.S. Environmental Protection Agency (EPA) commissioned the screening of 309 pesticides, herbicides, fungicides, and other chemicals of interest in a series of high-throughput screening assays called ToxCast (Dix et al. 2007; Knudsen et al. 2011). One of the targets tested in Phase I of ToxCast was PPARγ, and the screening commissioned by the U.S. EPA led to the identification of a group of chemicals with potential activity on PPARγ. We retested the top 20 most potent PPARγ activators identified in ToxCast for their ability to activate PPARγ using transient transfection assays in COS-7 cells and identified several as being bona fide PPARγ activators.

We selected the imidazole fungicide triflumizole (TFZ) for further study because it is a PPARγ activator and is widely used on food crops, particularly green leafy vegetables (U.S. EPA 2009). Although little is known about actual human exposure levels, 56,231 lb of TFZ were used in California alone in 2009. We tested TFZ for its ability to induce adipogenesis at biologically relevant concentrations using 3T3-L1 preadipocyte and MSC-based *in vitro* differentiation assays. TFZ induced adipogenesis *in vitro* in both cell types and promoted adipogenic gene expression in 3T3-L1 cells and in MSCs at low nanomolar concentrations. These effects were blocked by the specific PPARγ antagonist T0070907, establishing that TFZ exerts its effects through PPARγ. Administration of TFZ to pregnant CD-1 mice during gestation at approximately 400-fold below the established no observed adverse effect level (NOAEL) led to increased adipose depot weight and promoted adipogenic gene expression in the MSC compartment while reducing the expression of bone markers in the prenatally exposed male offspring. We infer that TFZ is likely to act as an obesogen *in vivo*.

## Materials and Methods

*Animal experiments.* Male and female CD1 mice (8 weeks of age) were purchased from Charles River Laboratories International Inc. (Wilmington, MA), housed in microisolator cages in a temperature-controlled room (22–24°C) with a 12-hr light, 12-hr dark cycle, and provided water and food (standard low-fat diet for rodents RMH 2500; Purina Mills, Richmond, IN) *ad libitum*. All animals were treated humanely with regard for alleviation of suffering, and all procedures were approved by the Institutional Animal Care and Use Committee of the University of California, Irvine. For prenatal chronic chemical exposure, dimethylsulfoxide (DMSO) (0.1%), rosiglitazone (ROSI) (0.5 µM), or TFZ (0.1, 1.0, or 10.0 µM) were supplemented in the drinking water during breeding and throughout pregnancy. Carboxylmethyl cellulose (CMC) at 0.5% was added to the water to increase the solubility of test chemicals, and control animals received water containing 0.5% CMC and DMSO vehicle. After the pups were born, normal filtered tap water was provided. The pups were kept together with their dams after birth and weaned at 3 weeks of age. Experimental mice were weighed and sacrificed at 8 weeks of age, and adipose tissues (epididymal fat for male and ovarian fat for female, retroperitoneal fat, and subcutaneous fat) were collected, weighed, and used for MSC production.

*Transfection.* The vectors pCMX-GAL4 and pCMX-GAL4-mPPARγ were previously described (Grun et al. 2006). Transient transfections were performed in COS7 cells as described by Chamorro-Garcia et al. (2012). Briefly, COS7 cells were seeded at 15,000 cells/well in 96-well tissue culture plates in 10% calf bovine serum. The following day, cells were transfected in Opti-MEM reduced-serum medium (all media and reagents from Invitrogen Life Technologies, Grand Island, NY unless noted otherwise) at approximately 90% confluency. One microgram of CMX-GAL4 effector plasmid was co-transfected with 5 µg tk-(MH100)_4_-luciferase reporter and 5 µg CMX-β-galactosidase transfection control plasmids using Lipofectamine 2000 reagent, following the manufacturer’s recommended protocol. After overnight incubation, the medium was replaced with Dulbecco’s modified Eagle medium (DMEM)/10% resin charcoal–stripped fetal bovine serum (FBS) (Tabb et al. 2004) plus ligands at concentrations indicated in the figure legends for an additional 24 hr before luciferase and β-galactosidase assays (Milnes et al. 2008). All transfections were performed in triplicate and reproduced in multiple experiments. Data are reported as fold induction over vehicle (0.1% DMSO) controls (mean ± SE) for triplicate samples (three biological replicates) and results were verified in multiple experiments.

*Cell culture.* 3T3-L1 cells were maintained in DMEM supplemented with 10% FBS, 2 mM l-glutamine, 100 U/mL penicillin, and 100 µg/mL streptomycin and differentiated as described previously (Li et al. 2011) using various concentrations of DMSO, ROSI, and TFZ. Briefly, cells were cultured until 2 days postconfluence, at which time the adipogenic induction cocktail MDI (IBMX, dexamethasone, and insulin) plus test ligands was added (Li et al. 2011). After 2 days, the medium was replaced with fresh medium containing test ligands and incubation continued for 5 additional days. For antagonist experiments, 1 µM T0070907 (Cayman Chemical, Ann Harbor, MI) was supplemented into the media every 12 hr. At the end of the experiment, cells were fixed and stained with Oil Red O to visualize lipid accumulation, or collected for RNA extraction, followed by QPCR [quantitative real-time reverse transcription-polymerase chain reaction (RT-PCR)] for gene expression analysis as described previously (Li et al. 2011). For spontaneous differentiation assays, 3T3-L1 cells were incubated at 2 days postconfluence in culture media supplemented with the indicated chemicals for 7 days.

Human white adipose tissue–derived MSCs were purchased from Lonza (Basel, Switzerland), cultured, and differentiated as described by Kirchner et al. (2010). Briefly, postconfluent cultures were treated with adipogenic or osteogenic induction cocktails together with test ligands or vehicle controls. Antagonist treatment was as noted above. Fourteen days (adipogenic) or 21 days (osteogenic) after differentiation was initiated, cells were stained with Oil Red O or collected for RNA extraction.

Mouse adipose tissue–derived MSCs were collected from the epididymal fat pads of male mice and cultured as described by Kirchner et al. (2010).

Oil Red O staining and quantitation of the lipid accumulation was previously described (Li et al. 2011). Briefly, lipid accumulation was assessed by measuring the percent of surface area in each well covered by Oil Red O–positive cells using ImageJ software (Rasband 2012). Data represent mean ± SE from triplicate treatments, with 6 images taken per well (*n* = 18 images total). {For representative photographs of adipogenesis assays, see Supplemental Material, Figures S1A, S2 (3T3-L1 cells), and S1B [human MSCs (hMSCs)] (http://dx.doi.org/10.1289/ehp.1205383).}

*QPCR.* Total RNA was isolated from cells and tissues using TRIzol reagent as recommended by the manufacturer (Invitrogen Life Technologies). Reverse transcription and QPCR were performed using Transcriptor reverse transcriptase and Sybr Green Master Mix (Roche Diagnostics Corp., Indianapolis, IN) (Li et al. 2011). The following genes were examined: *ADIPOQ* (adiponectin), *FABP4* (fatty acid binding protein 4), *FSP27* (fat-specific protein of 27 kDa), *LEP* (leptin), and *LPL* (lipoprotein lipase). [For details of the sequences of primers used for QPCR, see Supplemental Material, Table S1 (http://dx.doi.org/10.1289/ehp.1205383).]

*Statistical analysis.* Data are presented as mean ± SE. One-way ananlysis of variance (ANOVA) was used to determine the difference of means in relative mRNA abundance, staining, body weights, or adipose depot weights among TFZ treatment groups and negative control (DMSO). This was followed by a Dunnett’s post hoc test to ascertain statistical significance for each TFZ-treatment group compared with control (DMSO). The unpaired *t*-test was used to determine the significance of effects elicited by the positive control, ROSI relative to DMSO. Additional statistics were calculated for some experiments: One-way ANOVA with Bonferroni post hoc test was conducted comparing +T0070907 versus –T0070907 with each other. A *p*-value of < 0.05 was considered statistically significant. Statistical analysis used GraphPad Prism 5.0 (GraphPad Software Inc., San Diego, CA).

## Results

**Figure 1:**
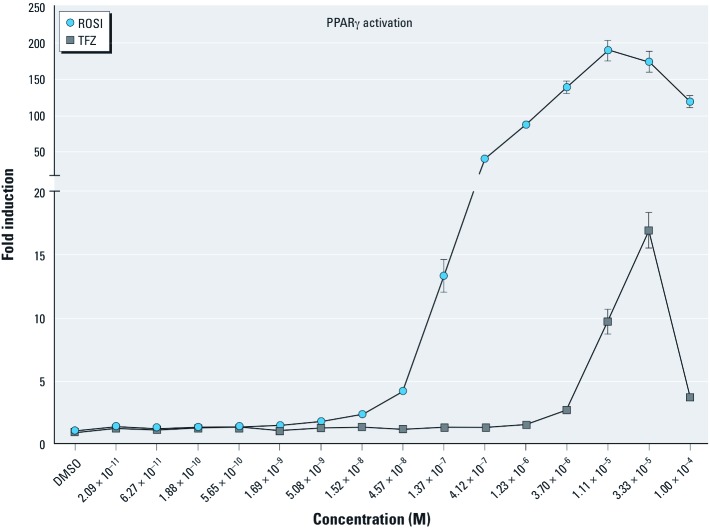
Activation of PPARγ by TFZ in transient transfection assays. The ability of a graded dose series of TFZ to activate GAL4-mPPARγ was tested in transiently transfected COS7 cells. TFZ and ROSI were tested in 3-fold serial dilutions from 10^–4^ M through 10^–11^ M. Cytotoxicity, as measured by decreased β-galactosidase activity was observed at 3.3 × 10^–5^M for both ROSI and TFZ. Data are depicted as fold induction over vehicle (0.1% DMSO) controls (mean ± SE); data points represent triplicate transfections (three biological replicates), and results were verified in multiple experiments.

*TFZ activates PPAR*γ. Many endocrine disrupting chemicals bind to and activate members of the nuclear receptor family, mimicking or interfering with the actions of natural lipophilic hormones (Diamanti-Kandarakis et al. 2009). For example, PPARγ is activated by the organotin compounds TBT and triphenyltin (Grun et al. 2006; Kanayama et al. 2005) in transient transfection assays. Because PPARγ is a key regulator of adipocyte gene expression and differentiation (Tontonoz et al. 1994), it is likely that other PPARγ activators will be obesogenic (Janesick and Blumberg 2011b). TFZ was identified as a PPARγ activator in the U.S. EPA ToxCast Phase I dataset (Knudsen et al. 2011), so we sought to verify this result by testing the ability of TFZ to activate PPARγ in transient transfection assays. TFZ activated PPARγ in a dose-dependent manner, although it was less potent than the pharmaceutical PPARγ activator ROSI in these assays (Figure 1).

**Figure 2:**
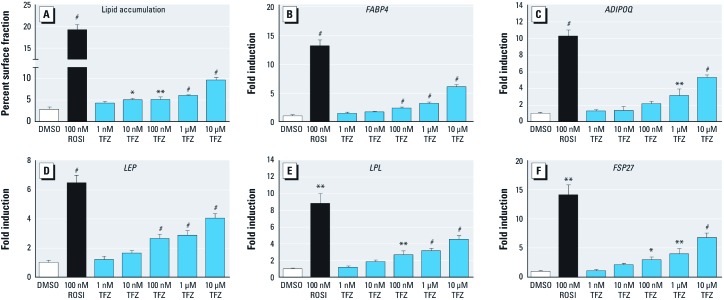
Effect of TFZ on adipogenesis in 3T3-L1 cells. The adipogenic effect of TFZ was tested in 3T3-L1 cells during MDI-induced adipocyte differentiation. Cells were treated with 0.1% DMSO (vehicle), 100 nM ROSI (positive control), or TFZ. Media were replaced every other day with freshly supplemented ligands. Seven days after differentiation was initiated, cells were fixed and stained with Oil Red O or processed for RNA extraction. (*A*) Lipid accumulation was assessed by measuring the percent of surface area in each well covered by Oil Red O–positive cells using Image J software. (*B–F*) Adipogenic gene expression determined by QPCR in cells collected on day 7 of differentiation. (*B*) *FABP4*, (*C*) *ADIPOQ*, (*D*) *LEP*, (*E*) *LPL*, and (*F*) *FSP27*. Data are presented as fold induction (mean ± SE) relative to DMSO vehicle for triplicate samples (three biological replicates), and results were verified in multiple experiments. One-way ANOVA was conducted for TFZ treatment groups and DMSO, followed by Dunnett’s post hoc test. Unpaired *t*-test was conducted for ROSI versus DMSO. *p < 0.05, **p < 0.01, and ^#^*p* < 0.001 compared with DMSO vehicle.

*TFZ increases adipogenesis in cell culture models.* Based on its ability to activate PPARγ, we hypothesized that TFZ would induce adipogenesis in the 3T3-L1 preadipocyte model previously utilized to characterize environmental obesogens (Chamorro-Garcia et al. 2012; Grun et al. 2006; Li et al. 2011). 3T3-L1 cells were treated with a concentration series of TFZ, 0.1 µM ROSI, or vehicle control [Figure 2A; and see Supplemental Material, Figure S1A (http://dx.doi.org/10.1289/ehp.1205383)]. In comparison to its modest ability to activate PPARγ in transfection assays, TFZ induced adipogenesis at concentrations as low as 10 nM (Figure 2A) with corresponding increases in the induction of adipogenic gene expression (Figure 2B–F). Although it did not elicit an equivalent response to 100 nM ROSI, 100 nM TFZ consistently induced adipogenesis and adipogenic gene expression (Figure 2). Some PPARγ activators (e.g., ROSI, TBT) can induce differentiation of 3T3-L1 cells without pretreatment with the adipogenic cocktail MDI (Grun et al. 2006). We found that TFZ enhanced adipogenesis in 3T3-L1 cells in the absence of MDI and induced adipogenic target genes such as *FABP4* and *ADIPOQ* (see Supplemental Material, Figure S2).

**Figure 3:**
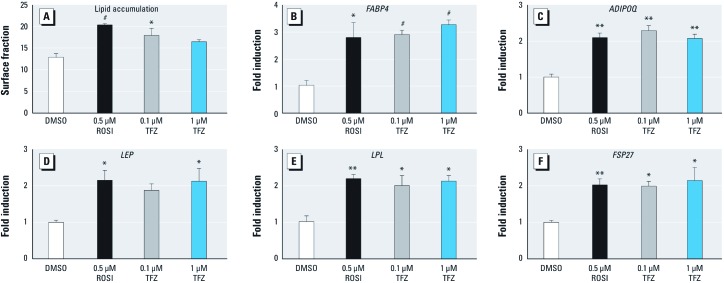
Effect of TFZ on adipogenesis in human white adipose tissue–derived MSCs. Adipogenesis was induced in hMSCs by adipogenic cocktail for 14 days in the presence of 0.1% DMSO (vehicle), 0.5 µM ROSI (positive control), or TFZ at 0.1 µM or 1 µM. (*A*) Lipid accumulation is shown by Oil Red O staining in hMSCs after 14 days of differentiation and quantified by measuring the percentage of surface area with lipid-laden adipocytes. (*B–F*) Adipogenic gene expression was determined by QPCR. (*B*) *FABP4*, (*C*) *ADIPOQ*, (*D*) *LEP*, (*E*) *LPL*, and (*F*) *FSP27*. Data presented are fold induction (mean ± SE) relative to DMSO and represent triplicate samples (three biological replicates), and results were verified in multiple experiments. One-way ANOVA was conducted for TFZ treatment groups and DMSO, followed by Dunnett’s post hoc test. Unpaired *t*-test was conducted for ROSI versus DMSO. *p < 0.05, **p < 0.01, and ^#^*p* < 0.001 compared with DMSO.

We previously showed that obesogens such as TBT (Kirchner et al. 2010) and bisphenol A diglycidyl ether (BADGE) (Chamorro-Garcia et al. 2012) could induce adipogenesis in MSCs derived from white adipose tissue or bone marrow. MSCs, and their more lineage-restricted derivatives, give rise to adipocytes *in vivo*, hence these cells are excellent models for studying adipogenesis (Cristancho and Lazar 2011; Rosen and MacDougald 2006). We tested whether TFZ could induce adipogenesis in human white adipose tissue–derived MSCs and found that 100 nM TFZ induced lipid accumulation (Figure 3A) and adipogenic gene expression (Figure 3B–F) to comparable levels induced by 500 nM ROSI. Taken together, these results indicate that TFZ activates PPARγ and induces adipogenesis in MSCs and in preadipocytes.

**Figure 4:**
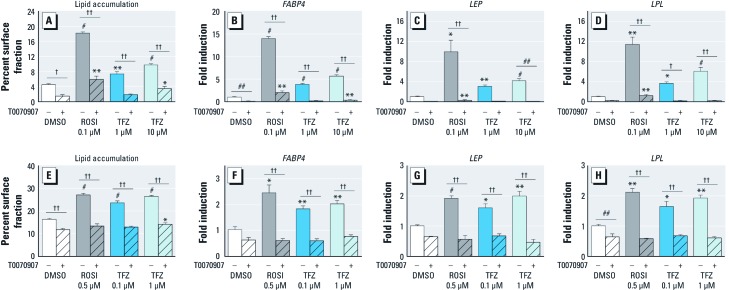
Effect of PPARγ antagonist T0070907 on TFZ-induced adipogenesis. (*A–D*) 3T3-L1 cells differentiated into mature adipocytes by the addition of MDI were treated with DMSO, 0.1 µM ROSI, or 1 µM or 10 µM TFZ, in the presence or absence of 1 µM T0070907. (*A*) Cells were stained with Oil Red O, and lipid accumulation was quantified by ImageJ software. Data are presented as area fraction (mean ± SE). (*B–D*) QPCR analysis of RNA extracted from 3T3-L1 cells was used to assess the expression of adipogenic genes *FABP4* (*B*), *LEP* (*C*), and *LPL* (*D*). (*E–H*) hMSCs differentiated into mature adipocytes by MDII (IBMX, dexamethasone, insulin, and indomethacin) were treated with DMSO, 0.5 µM ROSI, 0.1 µM or 1 µM TFZ, in the presence or absence of 1 µM T0070907. (*E*) Lipid accumulation was quantified as described for *A*. (*F–H*) RNA was extracted from hMSCs and analyzed by QPCR. Data are presented as fold induction (mean ± SEM) compared with DMSO for triplicate samples (three biological replicates) and results were verified in multiple experiments. One-way ANOVA was conducted for TFZ treatment groups and DMSO, followed by Dunnett’s post hoc test. Unpaired *t*-test was conducted for the positive control (ROSI) versus DMSO, and one-way ANOVA was conducted for all groups, followed by Bonferroni post hoc test comparing +T0070907 versus –T0070907. *p < 0.05, **p < 0.01, and ^#^*p* < 0.001 compared with DMSO. ^##^*p* < 0.5, ^†^*p* < 0.01, and ^††^*p* < 0.001 for +T0070907 compared with –T0070907.

*TFZ-induced adipogenesis is blocked by the PPAR*γ *antagonist T0070907.* We previously reported that PPARγ antagonists significantly diminished the adipogenic effects of TBT and of ROSI (Kirchner et al. 2010; Li et al. 2011), but not BADGE (Chamorro-Garcia et al. 2012), in 3T3-L1 cells and white adipose tissue–derived MSCs, indicating that some obesogens, but not others, act through PPARγ. We tested the effect of PPARγ inhibition on TFZ-induced adipogenesis in both 3T3-L1 cells and in hMSCs and found that T0070907 strongly inhibited adipogenic induction by MDI, ROSI, and TFZ in both cell types (Figure 4A,E). Furthermore, T0070907 treatment abolished the induction of adipogenic markers such as *FABP4*, *LEP*, and *LPL* (Figure 4B–D, F–H). These data suggest that the TFZ induces adipogenesis in both 3T3-L1 cells and MSCs through a PPARγ-dependent mechanism.

**Figure 5:**
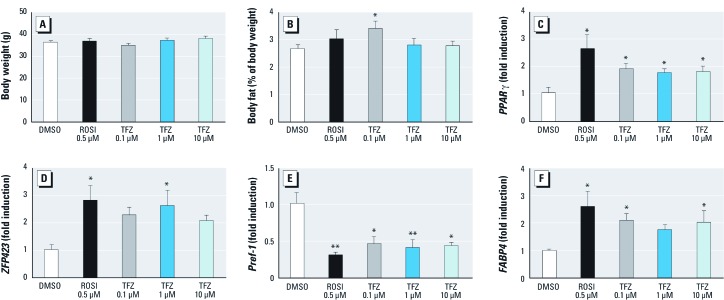
Effect of prenatal TFZ exposure on body weight, adiposity, and MSC programming in mice. Groups of three pregnant female CD1 mice were exposed to DMSO, ROSI, or TFZ via the drinking water; resulting male offspring were sacrificed at 8 weeks of age, and fat pads (epididymal, retroperitoneal, and subcutaneous) were collected and weighed. The numbers of exposed F1 offspring were as follows: DMSO, 17; ROSI, 14; 0.1 µM TFZ, 11; 1 µM TFZ, 15; and 10 µM TFZ, 14. (*A*) Body weight and (*B*) total fat depot weights were normalized to body weight and are expressed as the percentage of adiposity. (*C–F*) White adipose–derived MSCs were collected from mice; cells from mice derived from a single litter were pooled (three litters per treatment) and cultured until confluence. RNA was extracted and analyzed by QPCR; expression of *PPAR*γ (*C*), *ZPF423 *(*D*), *Pref‑1* (*E*), and *FABP4 *(*F*) was normalized to the housekeeping gene *36B4*. Data are expressed as fold change (mean ± SE) relative to DMSO controls. One-way ANOVA was conducted for TFZ treatment groups and DMSO, followed by Dunnett’s post hoc test. Unpaired *t*-test was conducted for the positive control (ROSI) versus DMSO. *p < 0.5, and **p < 0.01 compared with DMSO.

*Prenatal exposure to TFZ increases fat depot size and programs mouse MSCs to favor the adipogenic lineage.* We previously showed that exposure to the pharmaceutical obesogen ROSI or the environmental obesogen TBT activated PPARγ and induced adipogenesis in cultured MSCs. Moreover, prenatal exposure to ROSI or TBT reprogrammed this MSC population to favor the adipocyte lineage at the expense of bone (Kirchner et al. 2010). These results suggested that PPARγ activators can reprogram the MSC compartment to favor the adipocyte lineage. TFZ activates PPARγ; thus, we hypothesized that prenatal TFZ exposure would lead to increased adipose depot mass and elicit reprogramming of MSCs to favor the adipocyte linage. We tested the effects of exposing female CD-1 mice to TFZ, ROSI, or vehicle controls in the drinking water beginning with mating and terminating at birth. At 8 weeks of age, mice were sacrificed and total fat tissues (gonadal fat, retroperitoneal fat, and subcutaneous fat) were collected, weighed, and then used for MSC preparation. No significant changes in body weight were noted at 8 weeks of age (Figure 5A). Intriguingly, the lowest dose of TFZ used, 100 nM, produced a significant increase in fat depot weight whereas higher doses did not (Figure 5B).

In contrast to the effects on fat depot weight, QPCR analysis of gene expression in MSCs revealed significant changes in the levels of important adipogenic markers. Expression of the adipogenesis inhibitor, *Pref-1* (adipocyte differentiation-associated protein-1/preadipocyte factor-1), was down-regulated in MSCs from ROSI- and TFZ-treated animals (Figure 5E). Expression of *PPAR*γ, (Figure 5C) and *Zfp423* (zinc finger protein 423) (Figure 5D), which regulates *PPAR*γ expression (Gupta et al. 2010), were up-regulated by ROSI at all doses of TFZ. *FABP4*, a direct target of PPARγ action (Burris et al. 1999), was also strongly up-regulated by ROSI and TFZ (Figure 5F). QPCR analysis of markers of osteogenic programming [alkaline phosphatase (ALP) and runt-related transcription factor 2 (Runx2)] revealed that this MSC population was deficient in precursors of the osteogenic lineage [see Supplemental Material, Figure S3, (http://dx.doi.org/10.1289/ehp.1205383)]. These results are consistent with our previous findings using ROSI and TBT, both of which activate *PPAR*γ (Kirchner et al. 2010). These data collectively indicate that prenatal TFZ exposure alters cell fate in the MSC compartment to favor the adipocyte lineage, at the expense of bone, as expected for a chemical that acts through PPARγ.

## Discussion

TFZ is an imidazole fungicide that inhibits ergosterol biosynthesis. It was intended for multiple foliar applications to control powdery mildew, blossom blight, leaf spot, botrytis blight, rust, and scab in many food and ornamental crops. There are no peer-reviewed studies on the toxicity or action of TFZ *in vivo*. One recent study showed that azole-type fungicides, including TFZ, inhibited constitutive activation of retinoic acid receptor-related orphan receptors α and γ (Kojima et al. 2012). The only information from *in vivo* studies are from toxicity studies performed for TFZ licensing (U.S. EPA 2001, 2002). TFZ is licensed for use in corn, barley, and wheat and a variety of fruits and vegetables (U.S. EPA 2009). Approved modes of use include as a soil drench, foliar spray, and chemigation. The LD_50_ (the dose at which 50% of tested animals die) for TFZ in rodents is 1.42 g/kg. No carcinogenic or mutagenic potential was observed in rats or mice (U.S. EPA 2001, 2002). *In utero* TFZ exposure in rats led to reproductive and developmental defects, including fetal death, decreased litter size, and pup viability (U.S. EPA 2001, 2002); however, little is known about the potential physiological effects of TFZ.

Here we identified TFZ as a ligand that activates PPARγ, which indicates that TFZ could be a potential obesogen. TFZ was a less potent activator of PPARγ in transient transfection assays, compared with ROSI. TFZ exposure robustly promoted adipogenesis in 3T3-L1 preadipocytes, inducing triglyceride accumulation and the expression of adipogenic markers at concentrations as low as 10 nM (Figure 2). We also found that TFZ could induce adipogenesis in human adipose tissue–derived MSCs. Whereas TFZ was much less active than ROSI in 3T3-L1 cells, it was comparable to ROSI in its ability to induce adipogenesis and the expression of adipogenic markers in MSCs (Figure 3). This suggests that the potency of TFZ in transfection assays is not fully predictive of its ability to induce adipogenesis. This could be due to a lower efficacy of TFZ than ROSI or to the longer period over which TFZ acts in the 7–14 day adipogenesis assays compared with the 2-day ligand treatment in transfected cells. The adipogenic effects of TFZ on lipid accumulation and the expression of adipogenic markers was abolished by co-treatment with the PPARγ-specific antagonist T0070907 (Figure 4). Therefore, we conclude that TFZ mediates adipocyte differentiation on both stem cells and preadipocytes by activating PPARγ.

In light of these *in vitro* results, we tested whether prenatal exposure to TFZ affected adipose depot size and the adipogenic commitment of MSCs *in vivo*. CD-1 mice were exposed to 100 nM, 1 µM, or 10 µM TFZ via drinking water throughout pregnancy, and the effects on body weight, adipose depot size, and gene expression in the MSC compartment were evaluated at 8 weeks of age in offspring. Neither TFZ nor ROSI elicited any change in body weight at 8 weeks of age (Figure 5A). This is likely due to the relatively young age of these animals because changes in body weight tend to be exacerbated during aging. Moreover, body weight may not be a good measure of obesity in rodents because the inconsistency between *in vitro* adipogenesis and *in vivo* body weight gain is common, and therefore more parameters should be considered, including fat mass and adipose tissue cellularity (Thayer et al. 2012). Because the data for TFZ toxicity *in vivo* are limited and largely unpublished, we cannot rule out the presence of higher dosage–mediated side effects, which might lead to the loss of adipose mass, as was seen with perinatal DES exposure (Newbold et al. 2007). Indeed, we found that the lowest dose of TFZ, 100 nM, elicited a significant increase in fat depot weight as normalized to total body weight (Figure 5B). Although higher doses of TFZ did not increase adipose depot weight, all doses of TFZ altered programming in the MSC compartment, leading to an increased amount of adipogenic gene expression (Figure 5C) and decreased levels of osteogenic markers [see Supplemental Material, Figure S3 (http://dx.doi.org/10.1289/ehp.1205383)].

We previously showed that *in utero* TBT exposure led to the higher expression of adipogenic markers in the MSC compartment and to an increased number of cells committed to the adipocyte lineage (Kirchner et al. 2010). This enhanced adipogenic capacity rendered these animals more likely to store excess energy in the form of fat and increased their susceptibility to obesity. Although we observed increased fat depot weights only in the 100-nM group, all groups of TFZ-treated animals showed increased expression of early adipogenic markers in the MSC compartment (Figure 5C), suggesting that all groups are predisposed to increased adiposity.

Our data raise the question as to whether the doses we have used *in vitro* and *in vivo* are relevant to likely human exposures. The NOAEL for acute maternal and developmental toxicity of TFZ in rodents is 10 mg/kg/day, whereas the NOAEL for chronic exposure in the two-generation reproduction study in rats is 3.5 mg/kg/day and an extrapolated (and recently adjusted upward) absolute NOAEL of 3.5 mg/kg/day has been established (U.S. EPA 2009). In comparison, mice in our experiments showed increased fat depot weight at the lowest dose of TFZ, 100 nM in the drinking water. Assuming 10 mL of water is consumed per day by a 40-g animal, this projects to an approximate daily intake of 8.6 µg/kg—more than 400-fold below the reported NOAEL. The chronic reference dose (RfD) and chronic population adjusted dose (cPAD) for TFZ set by the U.S. EPA are 15 µg/kg/day, whereas the EC acceptable daily intake is 50 µg/kg/day (European Commission 2010; European Food Safety Authority 2009). There are no data available on human exposures; however, considering that 56,231 lb of TFZ were used in California alone in 2009 and TFZ is widely used on food crops (U.S. EPA 2009), it is reasonable to infer that humans are exposed to TFZ at detectable and perhaps significant levels. The doses used in our study are likely within the range of exposures experienced by the human population.

## Conclusions

Our data support the conclusion that TFZ is an obesogen *in vivo*, acting through a PPARγ-dependent mechanism. TFZ activated PPARγ, induced adipogenesis in 3T3-L1 cells and in primary MSCs and this induction was blocked by co-treatment with the PPARγ antagonist T0070907. Prenatal TFZ exposure increased fat depot weight *in vivo* and reprogrammed the MSC compartment to favor the adipocyte lineage. These effects occurred at doses of TFZ that are below the established NOAEL in rodents and within the range of estimated human exposure. Based on these data, we conclude that TFZ is a novel obesogen *in vivo*. Future studies will be required to establish the lowest doses of TFZ able to induce adipogenesis *in vivo*. It will also be of great interest to know whether TFZ exposure elicits transgenerational effects and epigenetic modifications in MSCs and what levels of TFZ and its metabolites are found in human biomonitoring and whether these are associated with obesity.

## Supplemental Material

(291 KB) PDF
