# Supplementary material for: Triflumizole Is an Obesogen in Mice that Acts through Peroxisome Proliferator Activated Receptor Gamma (PPARγ)
Source: Environ Health Perspect. 2012 Oct 22;120(12):1720–6. doi: 10.1289/ehp.1205383 (PMC3548286; doi:10.1289/ehp.1205383)
Supplement: (291 KB) PDF [file ehp.1205383.s001.pdf]

Supplemental material - Triflumizole is an Obesogen in Mice that Acts through Peroxisome Proliferator Activated Receptor Gamma (PPARγ)

Xia Li[1], Hang T. Pham[1], Amanda S. Janesick[1], and Bruce Blumberg[1, 2]

[1]Department of Developmental and Cell Biology, University of California, Irvine, California, USA

[2]Department of Pharmaceutical Sciences, University of California, Irvine, California, USA

Table S1 – Primers used for QPCR analysis of gene expression.

**Mouse primers**		
Gene	Forward	Reverse
m36B4	AAGCGCGTCCTGGCATTGTCT	CCGCAGGGGCAGCAGTGG
mAdipoQ	GTTCCTCTTAATCCTGCCCA	CTCCTGTCATTCCAACATCTC
mALP	GGGACTGGTACTCGGATAACGA	CTGATATGCGATGTCCTTGCA
mFABP4	TCACCTGGAAGACAGCTCCT	AAGCCCACTCCCACTTCTTT
mFSP27	CTGGAGGAAGATGGCACAATCGTG	CAGCCAATAAAGTCCTGAGGGTTCA
mLeptin	CCTGTGTCGGTTCCTGTG	CCTGTTGATAGACTGCCAGAG
mLPL	ACTCTGTGTCTAACTGCCACTTCAA	ATACATTCCCGTTACCGTCCAT
mPPARγ	GCGATTCCTTCACTGATAC	TCAAAGGAGTGGGAGTGGTC
mPref-1	CCTGGCTGTGTCAATGGAGT	CTTGTGCTGGCAGTCCTTTC
mRunx-2	TTTAGGGCGCATTCCTCATC	TGTCCTTGTGGATTAAAAGGACTTG
mZFP423	GAGGATACCCCTACGACGTG	GACTTGTCACGCTGTTCCTGTC

**Human primers**		
Gene	Forward	Reverse
hAdipoQ	TCCTCACTTCCATTCTGACTG	GGACCAATAAGACCTGGATCT
hb-actin	GACGGCCAGGTCATCACTAT	CGGATGTCAACGTCACACTT
hFABP4	AGCCCAACATGATCATCAGC	TTTCCATCCCACTTCTGCAC
hFSP27	CAGACAAGCCCTTCTTCCTG	TTATGGGAGAGGGACAGTGG
hLeptin	GGCTTTGGCCCTATCTTTTC	GGATAAGGTCAGGATGGGGT
hLPL	AGGAGCATTACCCAGTGTCC	GGCTGTATCCCAAGAGATGGA

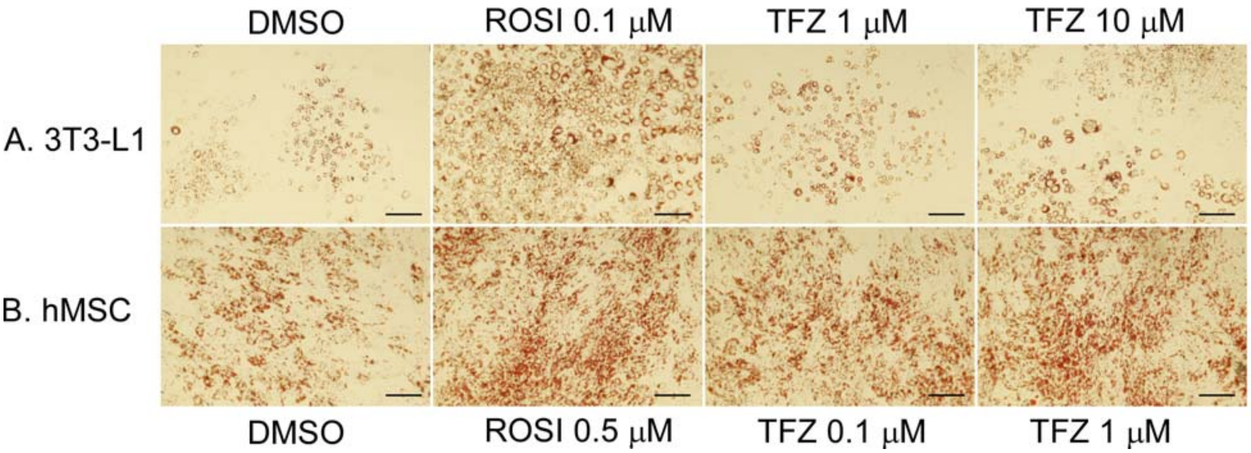

Figure S1. Oil Red O analysis of adipogenic induction in 3T3-L1 cells and MSCs.

Adipogenic induction and Oil Red O staining were performed as described in Methods. Representative pictures are shown for 3 different treatments. (A) 3T3-L1 cells (B) hMSCs. Scale bar = 200 μM

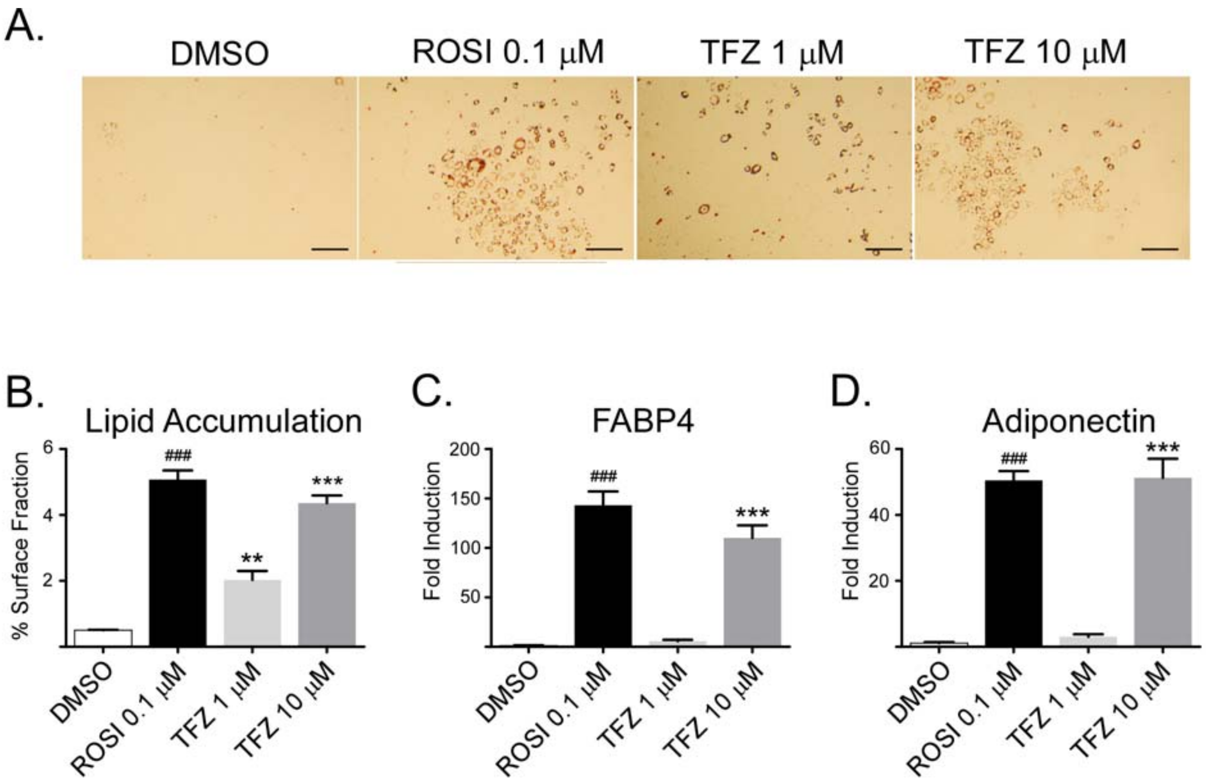

Figure S2. **Spontaneous differentiation in 3T3-L1 cells is induced by TFZ treatment.**

3T3-L1 cells were incubated in culture medium supplemented with DMSO, 0.1 µM ROSI, or TFZ at 1 µM or 10 µM for 7 days without treatment with the adipogenic cocktail MDI. (A) Cells were fixed and stained with Oil Red O for visualization of lipid accumulation. Representative pictures are shown for 3 different treatments. (B) Lipid accumulation was assessed by measuring surface area of culture plate covered by lipid-laden adipocytes using Image J software. (C, D) Cells were collected for RNA extraction, followed by QPCR analysis of the adipocyte specific markers, FABP4 and adiponectin. Data are presented as mean fold induction ± SEM relative to DMSO vehicle for triplicate samples. Data are representative for 3 independent experiments. Asterisks show significant differences compared with DMSO control. One-way ANOVA was conducted for TFZ treatment groups and DMSO, followed by Dunnett's post-hoc test: * $P < 0.5$, ** $P < 0.01$ and *** $P < 0.001$ compared to DMSO. Unpaired t-test was conducted for ROSI versus DMSO: # $P < 0.5$, ## $P < 0.01$ and ### $P < 0.001$. Scale bar in A = 200 µM

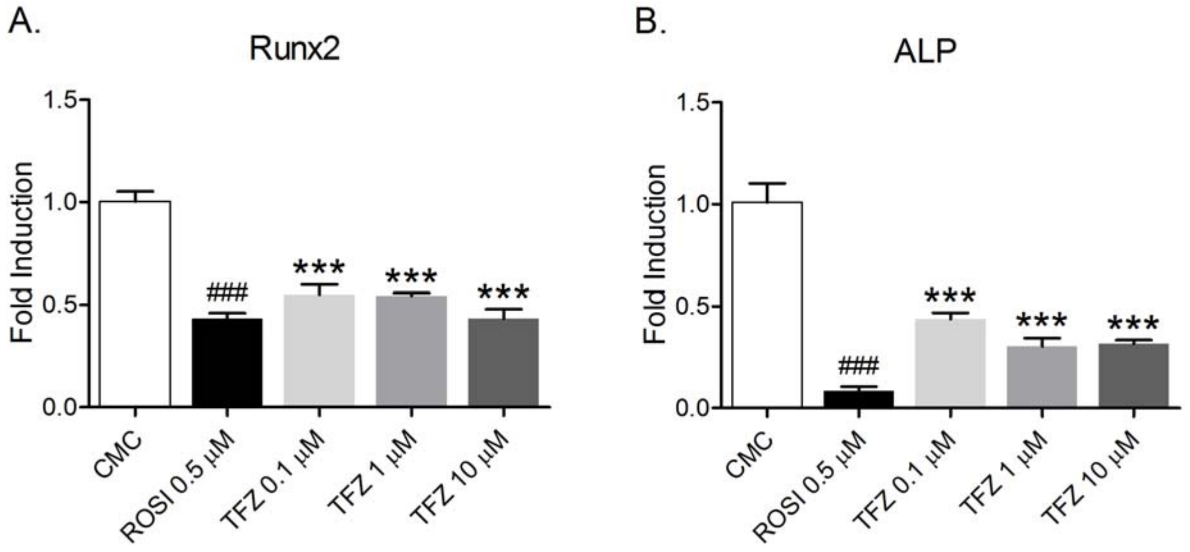

Figure S3. Effect of prenatal TFZ exposure on the osteogenic gene expression profile of mouse adipose derived MSCs.

Mouse MSCs were collected from white adipose tissue from TFZ exposed male mice and cultured till confluence. RNA was extracted for QPCR analysis of gene expression of the osteogenic genes, (A) Bone specific alkaline phosphatase (ALP) and (B) Runt related transcription factor 2 (Runx-2). Data are expressed as average fold change in expression mean ± SEM (n = 3 litters of mice) relative to CMC controls. One-way ANOVA was conducted for TFZ treatment groups and DMSO, followed by Dunnett's post-hoc test: * $P < 0.5$, ** $P < 0.01$ and *** $P < 0.001$ compared to DMSO. Unpaired t-test was conducted for ROSI versus DMSO: # $P < 0.5$, ## $P < 0.01$ and ### $P < 0.001$.
